# Supplementary material for: Increased adverse events following third dose of BNT162b2/Pfizer vaccine in those with previous COVID-19, but not with concurrent influenza vaccine
Source: PLOS Glob Public Health. 2023 Feb 17;3(2):e0001053. doi: 10.1371/journal.pgph.0001053 (PMC10021629; doi:10.1371/journal.pgph.0001053)
Supplement: Attachment — Submitted filename: Rebuttal.docx [file pgph.0001053.s002.docx]

**PLOS Global Health Response to Reviewers**

Dear editor,

Please find out responses to the revisions requested below:

**Journal Requirements:**

1. Please review your reference list to ensure that it is complete and correct. If you have cited papers that have been retracted, please include the rationale for doing so in the manuscript text, or remove these references and replace them with relevant current references. Any changes to the reference list should be mentioned in the rebuttal letter that accompanies your revised manuscript. If you need to cite a retracted article, indicate the article’s retracted status in the References list and also include a citation and full reference for the retraction notice.

**We have reviewed references and provided them in a consistent style/format**.

2. Please send a completed 'Competing Interests' statement, including any COIs declared by your co-authors. If you have no competing interests to declare, please state "The authors have declared that no competing interests exist". Otherwise please declare all competing interests beginning with the statement "I have read the journal's policy and the authors of this manuscript have the following competing interests:"

**We have no competing interests and have added the above statement as requested**.

3. Please amend your detailed Financial Disclosure statement. This is published with the article. It must therefore be completed in full sentences and contain the exact wording you wish to be published.

**We have provided a Financial Disclosure statement.**

a. State the initials, alongside each funding source, of each author to receive each grant.
b. State what role the funders took in the study. If the funders had no role in your study, please state: “The funders had no role in study design, data collection and analysis, decision to publish, or preparation of the manuscript.”
c. If any authors received a salary from any of your funders, please state which authors and which funders.

If you did not receive any funding for this study, please simply state: “The authors received no specific funding for this work.”

**We have amended the financial disclosure statement as suggested above.**

4. Please provide separate figure files in .tif or .eps format only and remove any figures embedded in your manuscript file. Please also ensure that all files are under our size limit of 10MB.

For more information about figure files please see our guidelines:

https://journals.plos.org/globalpublichealth/s/figures 
<https://journals.plos.org/globalpublichealth/s/figures#loc-file-requirements>

**We have now provided both figures in TIF format and included them in the body of the manuscript.**

5. We have noticed that you have uploaded Supporting Information files, but you have not included a list of legends. Please add a full list of legends for your Supporting Information files after the references list.

**We have now included a title for the supplementary data file.**

6. In the online submission form, you indicated that "All raw data will be made available upon request to the corresponding author". All PLOS journals now require all data underlying the findings described in their manuscript to be freely available to other researchers, either 1. In a public repository, 2. Within the manuscript itself, or 3. Uploaded as supplementary information.
This policy applies to all data except where public deposition would breach compliance with the protocol approved by your research ethics board. If your data cannot be made publicly available for ethical or legal reasons (e.g., public availability would compromise patient privacy), please explain your reasons by return email and your exemption request will be escalated to the editor for approval. Your exemption request will be handled independently and will not hold up the peer review process, but will need to be resolved should your manuscript be accepted for publication. One of the Editorial team will then be in touch if there are any issues.

**All data underlying the findings in our manuscript is available in the supplementary data file provided.**

**Reviewer #1: Reviewer Comments:**
The authors have a grandiose study, very important to health, as it tries to shade light on the implication for covid-19 vaccine uptake and immunization protocols. The study is very promising, but some minor points must be reviewed before possible publication.

**Thank you, we appreciate your feedback regarding the impact of our study.**

Abstract: The authors in the present study had a sample size of 534 HCW who were enrolled in the study: The authors should be specific how many individuals were analysed separately for AEs following all three doses of BNT162b2/Pfizer vaccine and those were involved in the concurrent seasonal influenza immunisation.

**This has now been added**

Methods:
The authors needs to provide ethical approval number for this study
The authors should clearly demonstrate how they arrived at sample size of 534 (show sample size calculation)

**No *a-priori­* sample size calculation was possible in this case, as the study was purely observational and retrospective. Our intention was to simply document the incidence of Adverse Events (AEs) in recipients of the third dose of covid vaccine, plus/minus seasonal influenza vaccine. In addition, as this was the first time of co-administration there was no basis for knowing which proportion would be administered within/without the 7-day window, and so any sample size calculation would have been totally speculative. The sample reported here is the total sample that responded to the invitation to participate.**

Results
In line 156-157 the authors states that “A similar pattern of results was found for prolonged AEs, which were scored as total number of vaccine-associated AEs rated as moderate/severe, lasting >24 hours”. In the subsequent studies they could have performed correlation analysis between the adverse events within less than 24hrs and after prolonged period in order to paint a clear picture as to whether the development and increase in AEs is observed after long period of vaccination.

**Thank you for this interesting suggestion. The data we collected as to the occurrence of AEs in each category was simply categorical with a binary outcome (yes/no), and as such, a correlation analysis is not possible for this sort of data at the level of individual AEs. There is indeed a strong positive correlation between the total number of symptoms and the total number of prolonged symptoms, but we do not report this as it appears a trivial consequence of the fact that a prolonged symptom must have been a symptom first.**

Conclusion
Since the large sample size that was analyzed constituted 85% female, can we conclusively state that AEs witnessed during covid-19 vaccination programs may be gender specific?

**Despite the reported gender imbalance (almost certainly due to the nature of the sample – HCW are biased towards females due to the preponderance of females in nursing and care roles); there was no significant different in rates of occurrence of AEs, as we report in lines 112/113 and table 1. There are associations of age with AE occurrence (as we report) and in the analysis of covariance we performed age and gender as appropriate were included as co-variates (lines 145/146) to control for these factors.**

**Reviewer #2: Reviewer’s comment**
Introduction
(1)Line 41: Authors should indicate the exact AEs that were frequently reported
(2)Lines 43/46: It would be appropriate if authors differentiate or state the specific AEs reported following a single dose of BNT162b2/Pfizer vaccine and the events reported during ongoing COVID-19 symptoms vaccination with BNT162b2/Pfizer 47 vaccine after second dose.

**The occurrence and severity of the same list of AEs was requested for doses 1,2 and 3 plus influenza vaccine in the current study (and in our two previous publications in this area – please see Raw et al 2021; 2022). Previous publications, as cited, demonstrate that the same cluster of AEs occur with each dose of vaccine albeit perhaps with lower frequency with repeated doses. The standard AEs investigated are those suggested by the FDA Toxicity Grading Scale as indicated in line 73.**

Methods
(1)Lines 78-80: Provide the ethics approval reference number

**We have added this.**

(2)The methodology employed in this students has some gaps that need to be addressed or filled.
a.Authors employed the use of email to invite responses. However, it was not clear how the respondents were selected, no clear inclusion and exclusion criteria were stated.

**We have added this information to the method section.**

b.Sample size was not predetermined.

**No *a-priori­* sample size calculation was possible in this case, as the study was purely observational and retrospective. Our intention was to simply document the incidence of AEs in recipients of the third dose of covid vaccine, plus/minus seasonal influenza vaccine. In addition, as this was the first time of co-administration there was no basis for knowing which proportion would be administered within/without the 7-day window, and so any sample size calculation would have been totally speculative. The sample reported here is the total sample that responded to the invitation to participate..**

c.The use of emails did not allow authors to verify the AEs being described. How were you able to verify these events?

d.Was the questions structured or unstructured?

**Structured – mainly multiple choice. Added to line 73.**

e. Were there avenue for follow up, if you required further information?

**Due to anonymity, this was not possible.**
f. The use of emails did not allow anonymity. In this case, how did you ensured anonymity? – the survey was online as stated in line 71

**In response to items c -f: There may have been some minor confusion here as to the nature of the study. This was a purely observational retrospective study of the occurrence of vaccine AEs in HCWs. Participation was invited by an email, but data collection took place using an anonymous browser based online questionnaire which entailed asking for no personally identifiable data and so was anonymous by design. All questions were of multi choice (often binary) response format (therefore “structured”) and data analysis was of the coded outcomes only. There was no possibility of follow up as this was an anonymous study. No verification of occurrence of AEs was possible as this was a self-report study but the very similar prevalence and reported patterns of AEs to our previous published work in this area are reassuring that participants are reporting their symptom occurrence with a reasonable degree of accuracy**

Results
(1)Lines 99/101: Avoid beginning a sentence with a number. Please revise
(2)Line 101: 144 HCWs (29.3%) did not indicate time interval. This seems a flaw in the design of the questionnaire. Authors should have envisaged this, even at the design stage.

**It is unfortunate that such a large number of participants did not provide this information. Ethical imperatives mean that at any point in a study participants may or may not disclose information as they wish and so therefore at no point in the online survey was a response forced. It is a well known issue particularly with online data collection that incomplete data may be collected. that Given that this was the first time the COVID and seasonal influenza vaccine had been co administered globally it is hard to understand how we could have controlled for participants not knowing the time interval between immunisations in particular other than by forcing a response to this question which would have not been ethically appropriate.**

Discussion
(1)Line 204: stud---study
(2)Our findings have implications for vaccine hesitancy and immunisation protocols. This was the concluding part of the abstract. The discussion section only has this on vaccine hesitancy ‘Given that vaccine safety plays a central role in people’s decision to accept a vaccination, our present work has implications for vaccine hesitancy’ It makes it very difficult to understand how this study could have implications on vaccine hesitancy and immunization protocols, in general. Please discuss the study to take care of this.

**Thank you for noticing this omission. We feel that our results should reassure people in general that (a) immunisation AEs to both COVID and influenza vaccine are generally mild and (b) co administration of immunisations within a short time period does not appear to significantly increase the incidence or severity of AEs. Whilst specific in this case to COVID and influenza vaccine it adds to the body of data supporting the safety of immunisation programmes worldwide and should be used to improve public confidence in such public health measures. This is already mentioned in line 232 and we will add a concluding sentence of this nature to the discussion section – see line 23.**

Reviewer #3: The authors are advised to include a statement about the type of study design that was employed.

**This was a retrospective observational and descriptive study of the incidence and prevalence of self reported AEs following COVID and seasonal influenza vaccine. Added to line 71.**

Page4 line 88: The authors should provide citation for the software JASPv0.16.3.0.

**This has now been added.**

Authors should avoid starting a sentence with abbreviations. There are also a couple of spelling errors in the discussion aspect of the manuscript, the authors are advised to revise for punctuation and spelling.

**This has been checked and errors corrected.**
